# Safe and sustainable by design of next generation chemicals and materials: SSbD4CheM project innovations in the textiles, cosmetic and automotive sectors

**DOI:** 10.1016/j.csbj.2025.03.022

**Published:** 2025-03-17

**Authors:** Mansoor Ahmad Bhat, Tanja Radu, Ignacio Martín-Fabiani, Panagiotis D. Kolokathis, Anastasios G. Papadiamantis, Stephan Wagner, Yvonne Kohl, Hilda Witters, Wouter A. Gebbink, Yentl Pareja Rodriguez, Giuseppe Cardelini, Roel Degens, Ivana Burzic, Beatriz Alfaro Serrano, Claudia Pretschuh, Eduardo Santamaría-Aranda, Elena Contreras-García, Judith Sinic, Christoph Jocham, Dror Cohen, Ze’evi Maor, Assaf Assis, Ondrej Panák, Uroš Novak, Sukriti Hans, Antje Biesemeier, Pau Camilleri, Fruela Pérez Sánchez, Thomas Arblaster, Nils Thonemann, Jeroen Guinée, Andrea Pipino, Onur Çelen, Hariprasad Alwe, Roland Drexel, Roland Welz, Florian Meier, Indre Piragyte-Langa Oliva, Ghada Tagorti, Barry Hardy, Milica Velimirovic

**Affiliations:** a School of Architecture, Building and Civil Engineering, Loughborough University, Loughborough, Leicestershire LE11 3TU, United Kingdom; b Department of Materials, Loughborough University, Loughborough LE11 3TU, United Kingdom; c NovaMechanics MIKE, Piraeus 18545, Greece; d Entelos Institute, Nicosia 1065, Cyprus; e Institute for Analytical Research, Hochschule Fresenius, Limburger Str. 2, Idstein 65510, Germany; f Fraunhofer Institute for Biomedical Engineering IBMT, Joseph-von-Fraunhofer-Weg 1, Sulzbach 66280, Germany; g Health Unit, Flemish Institute for Technological Research (VITO), Boeretang 200, Mol 2400, Belgium; h VITO-EnergyVille, Boeretang 200, Mol 2400, Belgium; i Wood K plus-Kompetenzzentrum Holz GmbH, Division Biobased Composites and Processes, Altenberger Strasse 69, Linz 4040, Austria; j BioNanoNet Forschungsgesellschaft mbH, Kaiser-Josef-Platz 9, Graz 8010, Austria; k Department of Sustainability and Advanced Materials, Footwear Technology Center of La Rioja (CTCR), Raposal 65, Arnedo, La Rioja 26580, Spain; l Ahava Dead Sea Laboratories Ltd., Dead Sea, Israel; m Department for Catalysis and Chemical Engineering, National Institute of Chemistry, Hajdrihova 19, Ljubljana 1000, Slovenia; n Advanced Instrumentation for Nanoanalytics group, Scientific Instrumentation and Process Technology Unit, Luxembourg Institute of Science and Technology (LIST), Belvaux 4422, Luxembourg; o Instituto Tecnológico del Embalaje, Transporte y Logística (ITENE), Carrer d′Albert Einstein, 1, ValenciaPaterna46980 Spain; p Institute of Environmental Sciences (CML), Leiden University, Einsteinweg 2, Leiden 2333 CC, the Netherlands; q Centro Ricerche Fiat, Strada Torino 50, Orbassano 10043, Italy; r KORTEKS A.Ş. Fethiye OSB, Ni̇lüfer, Bursa 16215, Turkey; s Tofwerk AG, Schorenstrasse 39, Thun 3645, Switzerland; t Postnova Analytics GmbH, street: Rankinestr. 1 in, Landsberg am Lech 86899, Germany; u Edelweiss Connect GmbH, Technology Park Basel, Hochbergerstrasse 60C, Basel 4057, Switzerland; v MatCh Unit, Flemish Institute for Technological Research (VITO), Boeretang 200, Mol 2400, Belgium; w Laboratory of Biochemistry and Biotechnology of the Skin, The Dead Sea & Arava Science Center, Masada, 86910, Israel

**Keywords:** Materials engineering, Nanotechnology, Chemical engineering, Nanoparticles, Chemical safety, Sustainability, Risk assessment, SSbD framework

## Abstract

The strategic objective of the Safe and sustainable by design of next generation chemicals and materials (SSbD4CheM) project is to develop screening and testing strategies for a variety of substances and materials to ensure safer and more sustainable products in line with the Sustainable Products Initiative. SSbD4CHeM is focusing on chemical safety using new approach methods, including *in vitro* studies without animal models and *in silico* tools. Additionally, it integrates environmental sustainability for the implementation of the Safe and Sustainable by Design (SSbD) framework including risk assessment and *ex-ante* life cycle assessment. New methods and models for safety and sustainability assessment along chemical, material and product life cycles will be developed, validated, and applied to three case studies, including biobased self-cleaning, water repellent, and antimicrobial treatments for textiles, nanocellulose as an additive in cosmetics, and microcellulose composites for the automotive industry. By employing a multidisciplinary strategy, SSbD4CHeM addresses key challenges in material innovation, ensuring regulatory compliance while reducing hazards to environmental and human health. The project will accelerate the development of next-generation sustainable materials, promoting industry innovation, regulatory progress, and improved consumer safety. Ultimately, SSbD4CheM aims to establish a new benchmark for the development of chemicals and materials that conform to safety and sustainability goals.

## Graphical Abstract

**Figure:**
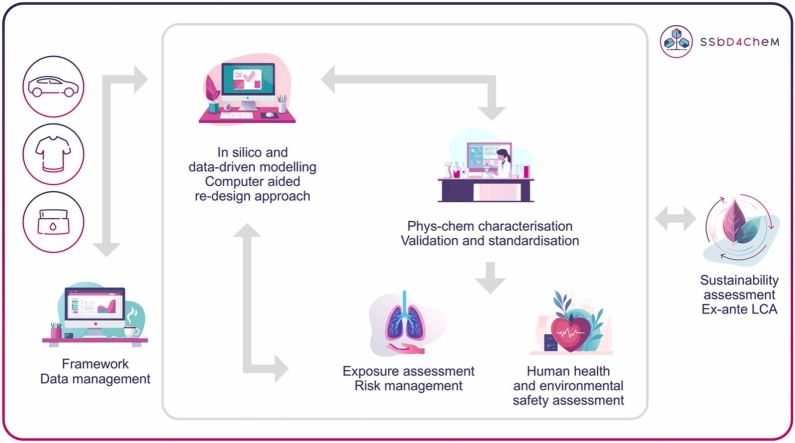

## 1 Introduction

The European Green Deal vision and objectives are set to put the European economy and society on a sustainable pathway to move towards climate neutrality, a circular economy, and a “zero pollution/toxic-free” ambition [1], [2], [3]. The key driver of this societal transformation is the transition to a safe- and sustainable-by-design (SSbD) approach, ensuring that chemicals, materials and products are designed, produced and used in a way that does not harm human health and the environment. The Chemicals Strategy for Sustainability has already started promoting the development of criteria for safe and sustainable-by-design products. Clearly, new methods and models supporting the strategy itself will have to be put in place. Designing safer and more sustainable chemicals, materials, products, and processes is crucial for achieving this transformation and re-establishing competitiveness for more sustainable products within the European manufacturing economy [2].

SSbD prioritises early supply chain efforts to create circular economy-compatible chemicals, materials, and products without harming human health or the environment. The approach balances functionality, circularity, climate neutrality, safety, social responsibility, and economic development and innovation throughout the lifespan of chemicals, materials, and products [4]. Chemicals serve a crucial role in our everyday lives, protecting our health and security and enabling innovation. They are present in almost all devices we use. Chemicals are key components of energy-efficient, low-carbon, and pollution-free technologies, materials, and products. Increased investment and innovation in the chemicals sector are crucial for safe and sustainable solutions, supporting green and digital transformations in our economy and society [2]. Chemicals with hazardous properties may affect humans and the environment. Some hazardous substances may cause cancer, harm the immunological, respiratory, endocrine, reproductive, and cardiovascular systems, reduce human resistance to vaccines, and increase disease susceptibility or disturb ecosystem populations [5], [6], [7].

To develop and deploy sustainable chemicals for green and digital transitions, it is crucial to increase innovation in the chemical industry and value chains and adopt the European Union (EU) chemicals policy to address hazardous chemical challenges more quickly and effectively [2]. This involves promoting safe and sustainable chemical use, minimising harmful substances, and phasing out harmful ones in consumer products [8]. The transition to chemicals that are safe and sustainable by design is not only a societal urgency and environmental concern but also a potential economic opportunity.

Developing new chemicals and materials requires compliance with various regulations and standards, which vary across regions and countries. This hinders the creation of a standardised SSbD. Even though access to information on chemicals, their toxicological properties and their presence in the environment has improved significantly through Registration, Evaluation, Authorisation and Restriction of Chemicals (REACH), the Information Platform for Chemical Monitoring (IPCHEM), the Life Cycle Data Network, and the efforts of the European Environment Agency (EEA), the lack of accessible data at present remains a challenge. Scientists, risk assessors, and risk managers require accurate and comprehensive information to make informed decisions about the risks associated with chemicals [9].

## 2 Project description

**Fig. 1:**
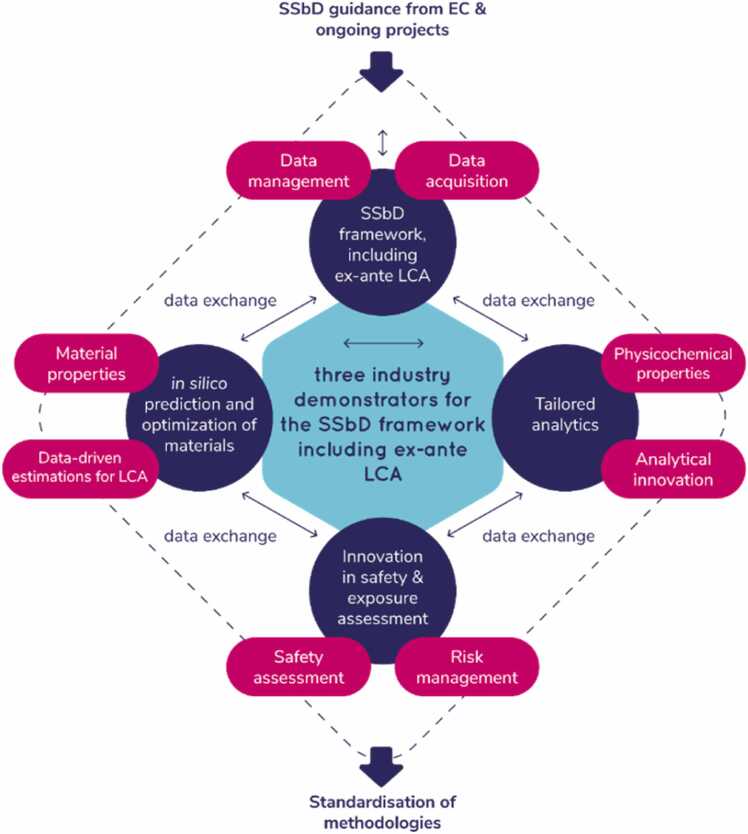
Overall SSbD4CheM workflow - Sustainable chemical design framework incorporating data management, collection, analytics, safety, and risk assessment. (SSbD: safe- and sustainable-by-design, EC: European Commission and LCA: Life cycle assessment).

The SSbD4CheM - Safe and sustainable by design of next generation chemicals and materials project aims to develop and promote best practices for safe and sustainable product and process design by bringing together stakeholders from industry, government, academia, and civil society. Through the demonstration of three case studies (textiles, automotive and cosmetics), the project seeks to meet the EU's strategic objectives for digital, enabling, and emerging technologies, sectors, and value chains. The objectives include the development of a comprehensive SSbD framework utilising new science-based approaches and innovative technologies to address potential hazards and risks. In addition, SSbD4CheM promotes the development of products and processes that are safe for humans and the environment while also being economically sustainable.SSbD4CheM is constructed as a toolbox (Fig. 1) with a collection of resources and tools designed to support the development of safe and sustainable products and processes. The toolbox includes a range of tools, guidance documents, databases, and other resources that can be used by stakeholders in industry, government, academia, and civil society to incorporate safety and sustainability considerations into their product and process design.

### 2.1 Project objectives

The implementation of SSbD principles in the pre-market phase of novel materials offers significant benefits such as improved product safety, minimised risks to human and environmental health, and addressed technical and scientific challenges while maintaining the material functionality. This approach aligns with Europe's transition towards a circular and low-carbon economy, as well as the European chemicals strategy for sustainability. Additionally, it ensures compliance with international guidelines and regulations (e.g., OECD, EC). To achieve this, the following SSbD4CheM objectives are set:

• Establishing an SSbD framework to facilitate the development of the next generation materials, including renewable composites, per- and poly-fluoroalkyl substances (PFAS)-free textile coatings, and nanocellulose additives.
• Proposing an efficient hazard screening tool that integrates *in silico* modelling and multicriteria analysis for assessing alternative chemicals, materials and products.
• Advancing an explorative *ex-ante* life cycle assessment (LCA) method, supported by molecular and data-driven modelling, to fill data gaps for novel materials and chemicals.
• Employing comprehensive chemical and (nano-)material assessment methods to evaluate products’ environmental safety, emissions, as well as consumer exposure limits.
• Adapting *in vitro* studies to enable non-animal models for adequate exposure scenarios.
• Promoting harmonisation and standardisation to ensure regulatory alignment and widespread adoption.
• Enhancing overall SSbD4CheM impact through stakeholder engagement, training, dissemination and exploitation.

### 2.2 Methodology overview

To address the key objectives of the project and further increase project performance, this work integrates safety and sustainability requirements into the development of new materials across three value chains: automotive (renewable-based lightweight composites in automotive interior parts), textile (plasma-applied bio-based coatings for water repellency and antimicrobial activity) and cosmetics (skin cosmetics products employing nanocellulose as an added value filler) value chains. To establish criteria for safe and sustainable design, these materials will undergo human health and environmental risk assessments, as well as life cycle assessments (LCAs). To support the computational and experimental work driving these assessments, specific requirements will be identified regarding the data, metadata, methods and protocols needed and act as a roadmap for the development of an SSbD framework. This framework is based on computational models (*in silico* and data-driven) (chapter 3.1), physicochemical characterisation (chapter 3.2), human and environmental hazard and risk assessment (chapter 3.3 and 3.4, respectively), as well as *ex-ante* LCA (chapter 3.5). The framework will be validated through the SSbD4CheM use cases to ensure its effectiveness and applicability.

### 2.3 SSbD4CheM’s consortium

The SSbD4CheM project has a highly interdisciplinary character, as well as promoting cross-sector collaboration as it brings together a diverse consortium from academia/research, small and medium enterprises, and industry (two large enterprises), combining scientific, technological, regulatory, societal, and business expertise. The project’s technical work involves a wide range of challenging tasks. It involves partners with complementary skills and a wide range of backgrounds including material developers, analytical chemists, computer scientists, (eco)toxicologists, process engineers, modelling experts, environmental and gender experts, and industrial end-users. This interdisciplinarity is apparent in the collaborative approach, which integrates expertise from different research fields, including: development of new analytical methods – Hochschule Fresenius (HSF), Postnova Analytics GmbH (PNV), Tofwerk AG (Tofwerk), Luxembourg Institute of Science and Technology (LIST), Vlaamse Instelling voor Technologisch Onderzoek (VITO)), data science (data bases and artificial intelligence – Edelweiss Connect GmbH (EwC), Entelos Institute Ltd (Entelos), Instituto Tecnológico del Embalaje, Transporte y Logística (ITENE)), engineering (development of new sustainable materials – Kompetenzzentrum Holz GmbH (WoodKplus), Asociación para la Promoción, Investigación, Desarrollo e Innovación Tecnológica de la Industria del Calzado y Conexas de La Rioja (CTCR), National Institute of Chemistry (NIC)), modelling and machine learning (e.g., *in silico* methods – NovaMechanics (NovaM), NIC), health/risk assessment (e.g., *in vitro* & ecotoxicological methods – Fraunhofer Gesellschaft zur Förderung der angewandten Forschung e. V. (FHG), VITO, Ahava Dead Sea Laboratories (AHAVA)), life cycle sustainability (implementation of SSbD framework – ITENE, Leiden University (ULEI)), risk management (Safe-by-Design – VITO, ITENE, Loughborough University (LU), BioNanoNet Forschungsgesellschaft mbH (BNN)), risk governance (standards, guidelines – Entelos, VITO, BNN), industrial end users (automotive – Centro Ricerche Fiat scpa (CRF), textile – Korteks Mensucat Sanayi ve Ticaret Anonim Sirketi (Kortex), cosmetics – AHAVA), as well as project dissemination, communication and exploitation (BNN, EwC). Every partner and its knowledge present a fundamental building block for this Research and Innovation project. Most consortium members have proven experience in collaborating on large-scale consortium projects, which has contributed to the establishment of good personal relationships based on a solid teamwork concept and a strong ethical commitment, as well as to the smooth running of operations throughout the project life cycle. Further information on team members can be found on the project website: https://www.ssbd4chem.eu/consortium/

### 2.4 Funding sources

The SSbD4CheM project has received funding from the European Union’s Horizon Europe research and innovation programme under grant agreement n° 101138475. UK participants in the SSbD4CheM project are supported by UKRI under grant number n° 10110559. CH participants in the SSbD4CheM project receive funding from the Swiss State Secretariat for Education, Research and Innovation (SERI). The EU Union’s Horizon research and innovation programme was designed to financially support research and innovation activities across various sectors and disciplines. The primary goal here was to tackle climate change, to help achieve the UN’s Sustainable Development Goals, and to boost the EU’s competitiveness and growth.

## 3 Impact

EU strategies and regulations, such as the Eco-Design for Sustainable Products Regulation and REACH, can benefit from new methods and data for chemicals and materials. All SSbD4CheM chemicals and materials must comply with REACH Article 33. The project will contribute to defining safety and sustainability criteria within the Sustainable Product Initiative, ensuring lower environmental impact and enhanced human health protection. To support regulatory alignment, methods and data will be made available for validation by networks such as EURL ECVAM and/or the OECD. The alternative screening methods developed through SSbD4CheM will undergo pre-validation, patenting, and commercialisation. Cooperation with partners will ensure Good Laboratory Practice (GLP) and Good Manufacturing Practice (GMP) compliance, facilitating qualification and market launch.

**Fig. 2:**
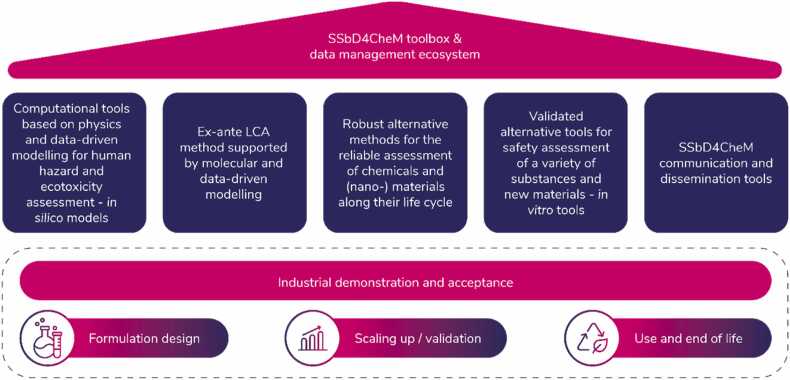
Framework of the SSbD4CheM toolbox and data management ecosystem for the safety and sustainability assessment of chemicals and materials. (SSbD4CheM: Safe and sustainable by design of next generation chemicals and materials and LCA: Life cycle assessment).

The methods and tools developed in the project will be accessible to industry and public authorities for comprehensive safety and sustainability assessment of chemicals and advanced materials (Fig. 2). This includes workshops, dataset analysis, validated computational workflows, web tools, and innovative 3Rs methods (Replacement, Reduction, and Refinement) and NAMs (New Approach Methodologies). These advancements will support the implementation of the SSbD framework, helping stakeholders to adopt safer and more sustainable practices.

Efforts will be made to harmonise and standardise methods and data by mapping and assessing current regulations and identifying gaps. SSbD4CheM will contribute to OECD or ISO-guided assessments and protocol standardisation, establishing contacts with relevant committees and stakeholders. SSbD4CheM will support the development of safe, sustainable materials, helping to phase out PFAS in the EU. It will use trusted parameters such as Life Cycle Costing (LCC) or LCA to demonstrate the sustainability of renewable composites and bio-based coatings, avoiding greenwashing. Finally, SSbD4CheM will boost research, development, and innovation in the EU by developing artificial intelligence (AI), Machine Learning (ML), and *in silico* workflows and web tools for the SSbD and data-driven design of renewable composites, PFAS-free coatings, and nanocellulose additives (Fig. 2).

### 3.1 Computer aided (re)design approach / Safe by material design

One of the key pillars of SSbD4CheM is the computer-aided (re)design approaches that can lead to Safe by Material Design (SbMD) approaches and strategies that can be applied as part of broader SSbD approaches. These approaches can be considered substantial drivers of data-driven innovation, especially under the context of the EU’s European Data strategy [10]. Integrating computer-aided (re)design and SbMD in current industrial processes and practices can lead to reductions in materials waste, enhance product efficiency, and, most importantly, more safe and sustainable materials for EU and international citizens. Publication of relevant computational tools and services, as SSbD4CheM is committed to doing, will assist with the EU’s strategic objectives regarding the digital single market, digital transformation, and green innovation [10]. Furthermore, they can support regulatory compliance, reduce the reliance on hazardous substances, and streamline the development of next-generation or the replacement of hazardous materials and chemicals. This can lead to a positive socio-economic impact from publicly funded projects, which can be achieved and increase consumer trust in sustainable products and digital processes.

As part of the SSbD4CheM framework, computer-aided (re)design approaches and SbMD aim to benefit from the integration of physics-based simulations and data-driven modelling, e.g., MML, to evaluate, predict, and optimise materials and chemical properties. In this way, the SSbD4CheM consortium employs a multi-scale computational framework to enhance material design using diverse *in silico* methodologies.

The SbMD approach within SSbD4CheM will be implemented using a combination of advanced physics-based simulations and data-driven models (e.g., Machine Learning), which will be used first to prioritise the experiments that will be conducted for the most promising materials (computer design approach). After the conduction of the experiments, the previously developed data-based models and the Force-Field parameters of the physics-based simulations will further be validated by their comparison with the experimental measurements and will be modified to reproduce the experimental results with high precision. Next, the modified data-based models and physics-based simulations will be further used to predict the most promising material structures for the textile, cosmetics, and automotive case studies (computer re-design approach). The produced models will be refined based on targeted experimental data produced within SSbD4CheM as part of an iterative update cycle.

**Fig. 3:**
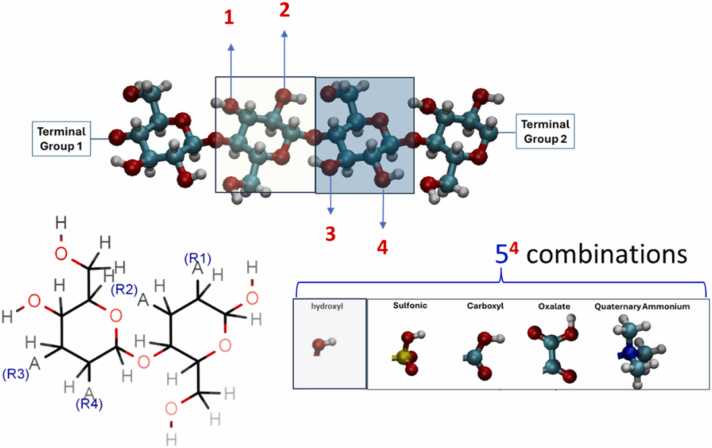
A Cellulose chain with the hydroxyl groups of two sequential monomers (top) and a cellulose dimer with its functional groups R1, R2, R3 and R4 (below, left) substituted with a range of groups (below, right). Carbon, Oxygen, Nitrogen and Hydrogen atoms are illustrated with light blue, red, blue and grey colors.

To begin with the computer design approach, data available in the literature are collected and used to train Machine Learning models. Concerning the cosmetics case study, skin sensitisation/irritation and acute dermal toxicity models are developed, which can predict the skin sensitivity of chemical compounds inside the limits of its applicability domain defined by the training dataset that has been used. These models can be used to investigate the effect of the substitution of the cellulose functional groups with other ones (Fig. 3) and are based on fingerprints and 2-D and 3-D descriptors of the chemical compounds. Investigation of their interactions with selected chemical compounds is applied through molecular simulations to reveal any hidden mechanism.

**Fig. 4:**
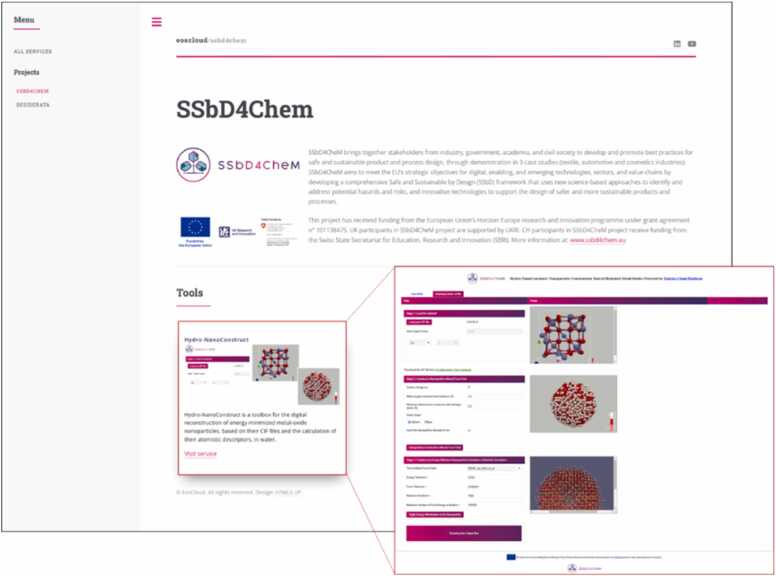
Eos Cloud Platform and its SSbD4CheM instance (https://eoscloud.entelos.eu/ssbd4chem.html), which hosts the Hydro-NanoConstruct tool for the digital construction of energy-minimised metal oxide nanoparticles in aquatic media at different pH and the calculation of their atomistic descriptors.

Concerning the automotive case study, a range of nanocomposite materials and their polymer networks are digitally constructed and will be compared with the properties of cellulose nanocomposite materials. To digitally build these nanocomposite systems, the Hydro-NanoConstruct tool has been developed (Fig. 4). The diffusion of volatile organic compounds through these polymer networks are also investigated using transition state theory and atomistics simulations. Concerning the textile case study, the effect of the surface coating of Poly Lactic acid and/or Polyethylene will be investigated through atomistic simulations for a range of precursors and the surface energy of the surface-modified textile materials, which is highly related to the contact angle of a water droplet on them.

### 3.2 Analytical methods for tailored requests

Basic data on the physical and chemical properties of SSbD4CheM materials are required as input for the SSbD assessment. For that purpose, existing analytical methodologies, including, e.g., targeted organic and inorganic chemical composition of the bulk, the spatial distribution of chemicals, morphology and shape retrieved by Fourier transformed infrared spectroscopy (FTIR) and electron microscopy (EM), as well as a set of physical characterisation methods (e.g., tensile strength). FTIR is widely used to identify functional groups and molecular structures, providing valuable information about the chemical composition of materials. EM, on the other hand, offers high-resolution images of material surfaces, enabling detailed analysis of morphology and shape. Physical characterisation methods, such as tensile strength testing, assess the mechanical properties of materials, which are critical for determining their suitability for specific applications. However, several physicochemical parameters required for SSbD assessment lack established methodologies, while others require substantial refinement. To address gaps in physicochemical characterisation and chemical exposure analysis, SSbD4CheM will develop innovative analytical methods tailored to non-uniform particles characterisation, emission profiling of volatile organic compounds (VOCs), identification of unknown compounds, including transformation products, composites characterisation and exposure quantification for risk assessment (Table 1). Newly generated physical-chemical data is prepared according to SSbD4CheM FAIR data templates and incorporated into the database.

**Table 1:** Methodologies to be developed in SSbD4CheM to determine relevant physicochemical material properties.

**Property**	**Methodologies to be developed**	**Relevant use cases**
Materials composition	Detection of unknown compounds using non-target analysis of materials using mass spectrometry and data evaluation schemes	Cosmetics
Non-uniform particles	Field-flow fractionation with emphasis on light scattering detection to characterise (nano-)particles with high aspect ratios	Cosmetics, Automotive
Volatile organic compounds	Target and non-target analysis using mass-spectrometry and efficient data evaluation schemes of composites and coatings	Textile, Automotive, cosmetics
Fiber characterisation and exposure in biological media	High resolution chemical imaging-based on Focused Ion Beam Secondary Ion Mass Spectrometry	Textiles, cosmetics

#### 3.2.1 Characterisation of non-uniform particles

New Field-Flow Fractionation (FFF) methods with light scattering detection are being developed to characterise non-uniform (nano-) particles with high aspect ratios [11], [12]. This methodology is essential for applications in both the cosmetics and automotive industries, where particle uniformity can significantly impact product performance and safety.

#### 3.2.2 VOCs in composites and coatings

Materials can emit toxic and odorous chemicals such as VOCs. To ensure occupational safety and product quality during the life cycle, trace levels of these chemicals need to be identified and quantified. Standard analytical protocols based on Gas chromatography/mass spectrometry analysis are time-consuming (up to 60 min per sample) and lack robustness. Time-of-flight mass spectrometry offers ultra-low (down to sub-ppt levels) detection of a large range of VOCs within only a few seconds. Depending on the ionisation sources, different chemical groups can be analysed. A more systematic understanding of the applicability of the sources for different material groups and test settings, as well as methods for quantification under industrial environments, is needed. Non-targeted analysis with soft ionisation sources allows the detection and possibly the identification of various unknown compounds [13]. Depending on the soft ionisation source, i.e. electrospray ionisation (ESI), Proton Transfer Reaction (PTR), Vocus Adduct Ionization Mechanism (AIM) [14] or Soft Ionisation by Chemical Reaction in Transfer (SICRIT), a wide range of compounds can be detected including ethylene, acetylene, most halocarbons, BTXEs Aromatics, Aldehydes: Formaldehyde, Acetaldehyde, Acrolein (2-methyl propenal) and organic acids. As proof of concept, the headspace of 12 different polyurethane (PU) based artificial leather and two foam samples were measured using PTR-ToF-MS [15], [16]. A real-time quantification method for VOCs emitted from raw materials and consumer goods in laboratory and industrial settings will be developed. This includes the selection of the most efficient ionisation source, methodology for quantification of relevant chemicals, identification of unknown compounds and an instrumental platform ready for industrial settings.

#### 3.2.3 Fiber characterisation and exposure in biological media

Currently, no suitable workflows exist to investigate the chemical composition of nanofibers, such as those in cosmetics or textiles, at high spatial resolution (better than 500 nm). Existing methods rely on standard analytical techniques without imaging capabilities or on molecular imaging methods limited to microparticulate or larger particle analysis. These methods are inadequate for analysing individual nanofibers within meshworks or detecting functional coatings and heavy metal impurities introduced during fabrication. Additionally, conventional chemical imaging tools, such as Scanning Electron Microscopy coupled with Energy Dispersive X-ray Spectroscopy and other techniques based on electrons, light, or X-rays, often lack the necessary sensitivity and lateral resolution for nanoparticles in exposed biological matrices. Comprehensive analyses often require multiple consecutive steps with differing sample preparation methods, which can complicate workflows. SSbD4CheM aims to develop innovative workflows for the physiochemical characterisation of textiles and fibres, both individually and in biological contexts (e.g., cells and tissues), using focused ion beam-based secondary ion mass spectrometry (FIB-SIMS) [17]. These workflows will address challenges such as the low conductivity of textile samples, the high topography of meshworks, and the low contrast of carbon-based fibres (e.g., cellulose, plastics) during risk assessment studies.

#### 3.2.4 Non-target analysis for safety and quality assessments

Quality and safety assessments usually rely on target analysis with a focus on the detection of known compounds and/or contaminants in materials and products. However, unknown compounds may be present in the material or product, either as inherent substances or as transformation products formed during the preparation and production process. While targeted analysis does not reveal their presence, untargeted approaches are required. Non-targeted analysis (NTA) is widely used in environmental monitoring, employing high-resolution mass spectrometry, possibly combined with gas chromatography or liquid chromatography, as well as with pyrolysis ovens where a large amount of unconsolidated data is produced. For the assessment of the data, efficient data reduction schemes are required. NTA has already been applied successfully in environmental sciences, elucidating the presence of unknown compounds such as transformation products in surface waters or in wastewater treatment plant effluents, but also in food analysis [18]. The SSbD4CheM project aims to develop a novel methodology for non-target analysis of advanced materials by using innovative instrumental setups, including ionisation sources and coupling techniques to detect unknown compounds. Additionally, NTA schemes of chemical compounds will progress regarding data reduction schemes.

### 3.3 Innovative tools for human health and environmental safety assessment

Over recent decades, the integration of New Approach Methodologies (NAMs) into human and environmental safety assessments has become increasingly critical. This shift aligns with evolving regulatory frameworks, emphasising animal ethics and the urgent need to reduce and replace traditional animal testing methods with alternative approaches to comply with the EU directive 2010/63/EU [19], [20]. NAMs encompass a broad spectrum of techniques, including adverse outcome pathways (AOPs) for predicting human disease outcomes, *in vitro* models, quantitative structure-activity relationship (QSAR) predictions, high-throughput screening assays, omics technologies, micro physiological systems (MPS) and AI-driven tools [21]. These novel approaches aim to cover mechanistic knowledge at the molecular or cellular level or key events in model systems that contribute to a better understanding of AOPs (human disease or environmental hazardous effects) and allow the classification of hazardous chemicals or materials.

The early implementation of validated NAMs in assessing the hazardous properties of novel materials or chemicals holds significant potential to optimise product design and manufacturing processes, also regarding time- and cost efficiency. The validation of the SSbD4CheM framework highlights its potential to replace animal testing. By integrating *in vitro*, *ex vivo*, and other innovative alternative models, this framework supports predictive toxicology through methods that simulate human physiology more accurately. As the SSbD4CheM framework evolves, its emphasis on advanced *in vitro* modelling ensures meaningful safety assessments by identifying potential hazards and risks. Validated NAMs contribute to designing safer, more sustainable products and processes, thereby aligning with global goals for ethical and environmentally conscious innovation.

In this way, the optimisation and pre-validation of advanced *in vitro*, *ex vivo* and zebrafish embryo models with their application in selected SSbD4CheM case studies will contribute to the implementation of validated NAMs in the SSbD toolbox.

**Fig. 5:**
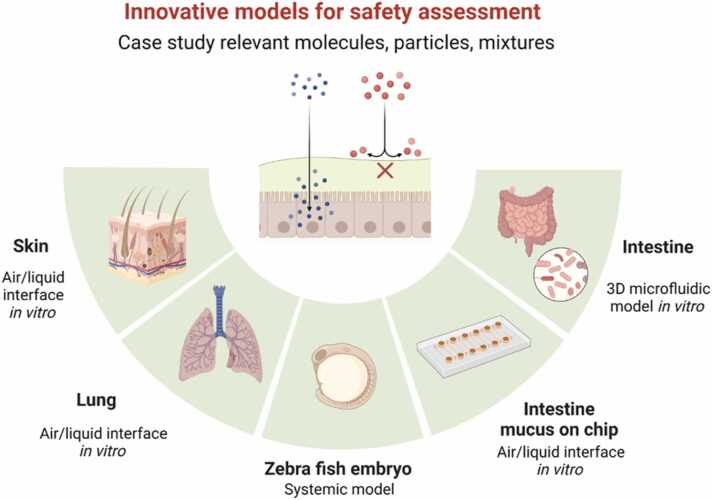
Innovative models for safety assessment within SSbD4CheM. Created with biorender.com.

SSbD4CheM will develop a suite of innovative complex models of the major tissue barriers that protect human health, placing them in a systems toxicology context (Fig. 5). Skin, lung, and gut barriers will be addressed together with the alternative model zebrafish embryo. The model design will allow the evaluation of low-dose chronic effects on human health and the environment. The predictive power of the new *in vitro* models will be tested against a matrix of characterised nanomaterials or chemicals. SSbD4CheM plans to translate promising new methods from the development phase (for advanced 3 R models) to achieve regulatory readiness with respect to draft guidelines and prototypes of model systems for future commercial development.

In the dermo-cosmetic field, the principles of the 3 Rs framework (Replacement, Reduction, and Refinement) have been implemented for decades to minimise the use of animal testing and reduce procedural cruelty. As a result, the testing of cosmetic ingredients and products on animals has been completely banned. However, the obligation to ensure the safety of cosmetic products and active ingredients remains unchanged [22]. This situation has further emphasised the need for validated alternative methodologies, for instance, topical application, which is emerging as a standard approach for assessing toxicity, efficacy, and safety. Furthermore, there is a need to establish comprehensive safety data sheets (SDS) for ingredients and determine No Observed Adverse Effect Levels (NOAEL) to accurately calculate the margin of safety (MOS) for cosmetic products [23].

Within the SSbD4CheM framework, three advanced *ex vivo* skin models will be developed to simulate specific stressors affecting healthy human skin. These models are designed to assess irritation, allergic responses, and UV-induced damage, with biomarker expression levels of key cytokines serving as indicators of skin response and hopefully will provide sufficient concrete data for regulatory evaluation in the future.

*In vitro* models of differentiated human induced pluripotent stem cells (hiPSC) will be generated according to dynamic bioreactor-based protocols [24], [25]. This approach does not require a carrier such as Matrigel and thus contributes to a reduction in the number of animals used for scientific purposes. Another 3R-compliant feature of this protocol is serum-free differentiation. This contributes to defined and standardised culture conditions and avoids sera of animal origin, such as fetal calf serum, as an important contribution to animal welfare. The *in vitro* lung barrier will be exposed to aerosolised particles, and cell viability, as well as specific lung markers will be quantified afterward.

The passage of orally ingested particles across the gastrointestinal barrier as well as their effects on molecular and cellular levels, will be investigated using microfluidic approaches. Gastrointestinal tissue (GIT) spheroids will be combined with a fluidic module and exposed to the test substances under fluidic conditions, followed by functionality investigation. In addition to the particle transport, another focus is on the interaction of the chemicals and particles with the mucus layer of the small intestine. For this approach, a fluidic mucus model will be improved and adapted. This model will also be optimised for long-term exposure and intended to be used for transport studies across the intestinal mucus – as the first barrier of oral exposure.

Next to cellular models, whole organism approaches, such as the zebrafish embryo, recognised as a non-animal model until 5 days post-fertilisation, can predict systemic effects. Through phenotypic observation for multiple toxicological endpoints, as e.g. teratogenicity, developmental neurotoxicity, and hepatotoxicity, this vertebrate embryo organism has been shown to be suitable for early prediction of human or mammalian toxicity [26], [27], [28], [29] and chronic environmental effects [30]. The embryo offers numerous advantages in a semi-throughput toxicity screening because of its size, fast development, and transparency, while it is a thoroughly studied embryological model for human disease and drug development, and large homology with the human genome [31]. Therefore, the latest omics approaches, e.g. RNA sequencing methods using the zebrafish embryo model, have shown promising results, which support mechanistic understanding of toxicological effects. For selected groups of hazardous chemicals, transcriptomic studies will be performed with the zebrafish embryo model that support phenotypic assessments related to known Adverse Outcome Pathways (AOP). Panels of biomarkers will be identified, aiming to categorise chemicals or prioritise them in the innovation cycle based on their mode of action and potential to predict human health or environmental hazardous effects.

### 3.4 Exposure assessment/risk management

Exposure and risk assessments play an important role in the evaluation of the safe use of chemicals under the REACH Regulation, and these assessments are incorporated within the SSbD Framework [32] when evaluating the implementation of new chemicals in products. Human and environmental exposure to chemicals can occur at all life cycle stages of the chemical/product, i.e., manufacturing of the chemicals, formulation, use of the product, and end-of-life. Within SSbD4CheM, a series of tools (including both experimental and *in silico* methods) will be used to identify particles and chemicals released from products. These tools will also be used to estimate releases, determine occupational, consumer, and environmental exposures, and for risk assessment, relevant to the three case studies (automotive, textile, and cosmetics).

The first step in assessing exposure is to identify emission sources and quantify the emissions. For example, within the textile case-study, the identification and releases of micro- and nanosized particles from different yarns and chemicals used for the water repellence and antimicrobial activity coatings will be monitored during various steps in the life cycle by using different analytical techniques. Standardised washing experiments [33] will be conducted to estimate releases of particles to wastewater, particle count, shape, size, and chemical composition will be determined using various appropriate analytical techniques. Emissions from coated fabrics will be performed in emission chamber experiments, to estimate releases to air as a potential exposure path for consumers. For the automotive case-study, VOC releases from car interior trims will be measured during and after the production process, as well as after artificial weathering to assess the potential consumer exposure (car drivers, passengers). These types of experiments will allow simulating real-life exposure at an occupational and/or consumer use setting under various conditions.

A novel approach of introducing *in vitro* toxicity assays into the emission test chambers will allow for directly combining exposure and adverse effect, leading to safety assessment of realistic scenarios. For relevant exposure scenarios within the three case-studies, where no experimental data can be generated within the project, *in silico* methods will be used to estimate human (occupations and consumer) and/or environmental exposure.

As an innovative approach, safe values will be derived from the *in vitro* testing of hazards, where the experimental *in vitro* dose causing (no) effect is translated to an *in vivo* dose for safe human exposure. This includes the use of physiologically based kinetic (PBK) modelling-based reverse dosimetry for the quantitative *in vitro* – *in vivo* extrapolation (QIVIVE) of *in vitro* effect values (EC50, NOEC) to equivalent doses [34].

The fate and behaviour of the chemicals in the environment will be assessed using *in silico* methods. Using data on physico-chemical properties and environmental fate properties, such as degradation and bioaccumulation, the fate of releases during relevant life cycle stages will be estimated resulting in predicted environmental concentrations in various environmental compartments. For both the human and environmental exposure assessments, measured or predicted exposure concentration will be compared to safe levels to assess potential risk. Performing these types of assessment when implementing the SSbD Framework ensures that the development of new chemicals/products poses a lower risk to human health and/or to the environment compared to existing products.

### 3.5 Life cycle sustainability assessment

Sustainable design principles give qualitative insight into opportunities to reduce the environmental and societal impacts of new chemicals and materials [32]. Unavoidably, trade-offs are encountered in the form of burden shifting, e.g., a solution to reducing use-phase impacts on climate change might increase resource acquisition impacts on eutrophication. LCA enables the systematic identification and quantification of such trade-offs, as well as providing further insight into design opportunities for impact reduction, thereby making a critical contribution to the innovation process [35], [36].

*Ex-ante* LCA is complicated by its prospective nature. As the early stages of development progress, an ongoing collaboration is required to understand where uncertainties lie, what degrees of freedom are available, and how experimental data and pilot testing might translate to conditions at market entry [37], [38]. Furthermore, the uncertain future context of the product calls for the implementation of broader forecasting tools [39], [40].

While the toolbox for prospective environmental assessment grows, these challenges are no less relevant when assessing social or economic sustainability. These perspectives offer insight into how these new chemicals affect societal factors like job creation, labour conditions, or affordability across the three key sectors studies in the project. This comprehensive framework for life cycle sustainability assessment will ensure that SSbD innovations meet environmental and socio-economic goals across diverse applications.

## 4 SSbD4CheM demonstrators – a practical implementation approach for SSbD principles

**Fig. 6:**
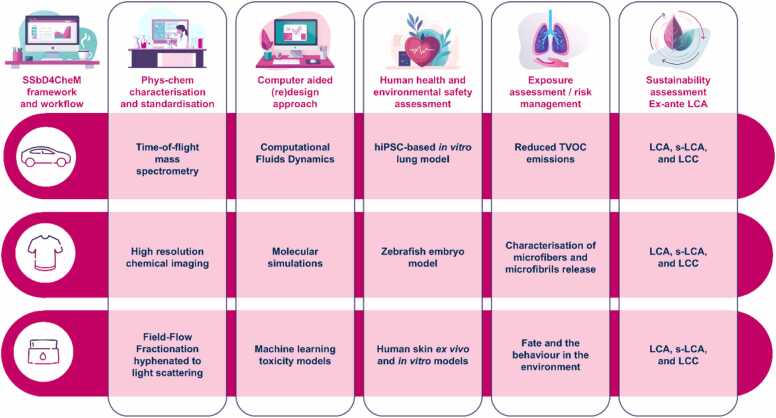
SSbD4CheM industrial demonstrators from automotive, textile and cosmetics sectors acting as safe and sustainable materials and products manufacturers, to provide: (i) material samples, (ii) industry insight, and (iii) application and validation of SSbD4CheM framework. (LCA: Life Cycle Assessment, s-LCA: Social Life Cycle Assessment, TVOC; Total Volatile Organic Compound, hiPSC: Human Induced Pluripotent Stem Cells).

To fully leverage the potential benefits that innovative renewable composites for automotive, PFAS-free coatings for textiles and sustainable additives like nanocellulose in cosmetics may offer, it is essential to prioritise addressing safety aspects in conjunction with sustainability considerations. Thus, the SSbD4CheM project incorporates this already in the design phase of SSbD4CheM materials, which can smoothen the R&D development process and save costs towards the market introduction of these materials (Fig. 6). By applying different steps of the Safe and Sustainable by Design (SSbD) framework, the SSbD4CheM project aims to integrate functionality/innovation with safety and sustainability considerations as early as possible in the innovation process at Technology Readiness Level (TRL3/4). Safety and sustainability concerns at TRL 3/4 address risks and environmental implications early in the innovation process. TRL 3 experimental proof-of-concept investigations confirm novel materials and chemicals' basic principles, whereas TRL 4 incorporates controlled laboratory validation. SSbD4CheM assures that materials are safe, functional, and environmentally friendly before moving to higher TRLs and commercial applications by incorporating SSbD techniques at these phases. This proactive strategy reduces risks, improves regulatory compliance, and speeds sustainable innovation market readiness. In parallel, sustainability entails incorporating ecological, social, and economic factors into the design and advancement of SSbD4CheM composites, coatings and additive materials and their related products and processes.

### 4.1 SSbD driven R&D for Renewable based lightweight composites in automotive interior parts

Until now, car interior trims have been made of plastics like polypropylene (PP) and filled with conflict minerals like talcum. For the development approach of more sustainable materials, we use recycled PP or PP made from renewable resources in combination with wooden fillers or fibrous cellulose powder as a main sustainable alternative to mined minerals like talcum powder. Compounds with reduced material density are in addition, lightweight materials, leading to reduced fuel consumption [41].

According to the SSbD approach, the following steps are considered in parallel during the R&D phase:

• Safety aspects during the material production, like potential dust inhalation from fine wooden fillers.
• A great focus is lying on the driver’s health aspects during the use phase of the car. It must be ensured completely that no hazardous substances are emitted from the material into the interior air of the vehicle. Different global standards and guidelines for such car interior VOC emissions are available for commercial plastic materials [42], but not for the developed cellulosic or wooden-filled lightweight composites.
• The assessment of the environmental and social sustainability of different filler types and the finished lightweight car interior product, as an ex-ante approach, enables the selection of more sustainable filler types already during the screening step of different material formulations according to their mechanical strength performance.
  Further, multiple criteria decision analysis (MCDA) [43] in combination with literature and database reviews, is an approach to support the final product design. This allows us to see the final decision as a compromise among different alternatives for the material composite formulation. The criteria include:
• ensure required material performance (mechanical strength)
• absence of chemicals classified as Group A and Level 1 substances minimised in the product
• avoided release of hazardous substances during the use phase
• an increased environmental and social sustainability by direct comparison with the benchmark product

### 4.2 SSbD-driven R&D on bio-based plasma coatings for antimicrobial and water-repellent textiles

Textile functionalisation allows the development or addition of new qualities to fabrics of different compositions, modifying their performance and broadening their range of uses. By applying specialised coatings, textiles can get advanced functionalities such as water repellency or antimicrobial properties.

Traditional coating processes, while beneficial for improving textile characteristics, have considerable environmental and health risks. For example, hydrophobicity is often accomplished using PFAS, which are harmful to both human health and the environment due to their persistence and bioaccumulation. Similarly, antimicrobial properties are frequently added to textiles using metals like silver. However, the use of these chemicals can lead to environmental problems due to leaching and toxicity. In addition to these issues, traditional coating methods like padding and exhaustion often require significant water consumption and substantial energy demand for drying and curing.

To address these challenges, the SSbD-driven R&D process focuses on developing bio-based, plasma-assisted coatings for textiles such as PET, recycled PET and PLA [44]. These innovative coatings are proposed as a sustainable alternative to traditional PFAS and silver-based treatments and are designed to:

• Provide antibacterial activity, improving hygiene and reducing the risk of contamination.
• Offer water-repellency, enabling a self-cleaning effect and enhancing durability.

Guided by an SSbD approach, the R&D of new textile coatings will focus on essential considerations that balance functionality with sustainability, ensuring high performance while minimising environmental impact. The key aspects addressed in this development include:

• Promoting sustainable practices by implementing dry plasma processes that lower the need for water and energy in textile treatment.
• Using bio-based chemicals (e.g. biobased acrylates) [45], [46] to replace fossil-based materials.

• Minimizing the use of chemicals by creating ultra-thin coatings in the nanometer to micrometer range.
• Lowering the environmental impact of the new coatings by conducting LCA studies that will help to identify key areas for impact reduction.
• Ensuring worker and user safety, with a particular focus on inhalation, skin, and oral exposure.
• Aiming to develop biodegradable textiles, ensuring they break down safely and reduce long-term environmental waste.

### 4.3 SSbD-driven R&D of skin cosmetics products employing nanocellulose as an added value filler

Nanocellulose, a material derived from natural cellulose fibers, has been gaining attention as a potential cosmetic ingredient, offering a sustainable alternative to conventional, non-nanocellulose forms [47]. Its unique physicochemical properties, including a high surface area-to-volume ratio, excellent moisture absorption and retention capabilities, and compatibility with a wide range of cosmetic ingredients, make it an attractive additive in cosmetic formulations [48]. However, the incorporation of nanomaterials into cosmetic products might be associated with regulatory and safety concerns, which are addressed by the REACH regulation with its revised annexes [22]. Therefore, the approval of nanomaterials for use in consumer products must be done by correctly implementing appropriate methods [49] that exclude animal testing.

One potential benefit of utilising nanocellulose in cosmetics is its sustainability, as it is a natural, renewable material. However, it's important to note that the production of nanocellulose itself can have environmental impacts, so it is important to consider the entire life cycle of the material.

In the SSbD-driven R&D process, our primary objective will focus on replacing non-nano ingredients with advanced nanocellulose additives while maintaining and enhancing the properties of four cosmetic products: facial cream, facial mud mask, base Dead Sea mud, and sun protection lotion. The main functions of nanocellulose additives are:

• Providing stability of the dispersion
• Regulate consistency
• Enhance texture and sensory (skin-feel) properties
  The nanocellulose additives being considered include cellulose nanocrystal (CNC), cellulose nanofibril (CNF) and bacterial nanocellulose (BNC). These nanocellulose additives vary in size, shape (morphology) and functional groups, which may influence their performance in cosmetic formulations.
  We aim to address the following aspects under the SSbD approach:
• Safety of a newly used advanced nanomaterial, especially on skin, by employing ex vivo and in vitro models to ensure consumer safety.
• Computational methods to assess skin toxicity of nanocellulose additives.
• Detailed physical-chemical characterisation of nanocellulose additives, including newly developed light scattering methods. Detailed characterisation is needed in different steps of SSbD and modelling tools.
• Environmental and socioeconomic impact of the new nanocellulose additives in comparison to the benchmark.

## 5 Summary and outlook

SSbD4CheM integrates hazard screening, risk evaluation, and sustainability assessments including LCA, LCC, and sLCA to comprehend material safety and sustainability, representing a significant innovation. To minimise dependence on traditional testing techniques and improve risk evaluation accuracy and efficiency, SSbD4CheM employs New Approach Methodologies (NAMs), *in silico* modelling, and non-animal-based toxicity assessments. These methods will promote the European Union's toxic-free, circular, and low-carbon economy goals by facilitating REACH and the Sustainable Products Initiative compliance. To spread its methodologies and tools across sectors, SSbD4CheM prioritises stakeholder engagement, industry cooperation, and regulatory alignment beyond its scientific contributions. The initiative promotes collaboration between academics, industry, policymakers, and regulatory authorities to accelerate the market introduction of sustainable, non-toxic industrial materials. The project's SSbD toolbox will help stakeholders integrate safe-by-design into material development, demonstrating the feasibility of developing chemicals and materials that are both useful and intrinsically safe for humans and the environment. By integrating advanced scientific methods with policy-oriented sustainability objectives, SSbD4CHeM will impact future research, industrial practices, and regulatory frameworks, fostering a more sustainable and responsible materials industry. Finally, SSbD4CheM will help industrialists and regulatory bodies while also equipping customers with safer, environmentally friendly goods, therefore fostering a healthier and more sustainable society.

## CRediT authorship contribution statement

**Mansoor Ahmad Bhat:** Writing – original draft, Writing – review & editing, Conceptualization, Investigation. **Tanja Radu:** Writing – review & editing. **Ignacio Martín-Fabiani:** Writing – review & editing. **Panagiotis D. Kolokathis:** Visualisation, Writing – original draft, Writing – review & editing. **Anastasios G. Papadiamantis:** Writing – original draft, Writing – review & editing. **Stephan Wagner:** Writing – review & editing. **Yvonne Kohl:** Writing – original draft, Writing – review & editing, Visualization. **Hilda Witters:** Writing – original draft, Writing – review & editing. **Wouter A. Gebbink:** Writing – original draft, Writing – review & editing. **Yentl Pareja Rodriguez:** Writing – review & editing. **Giuseppe Cardelini:** Writing – review & editing. **Roel Degens:** Writing – review & editing. **Ivana Burzic:** Writing – original draft, Writing – review & editing, Supervision. **Beatriz Alfaro Serrano:** Visualisation, Writing – review & editing. **Claudia Pretschuh:** Writing – original draft, Writing – review & editing. **Eduardo Santamaría Aranda:** Writing – original draft, Writing – review & editing. **Elena Contreras García:** Writing – original draft, Writing – review & editing. **Judith Sinic:** Writing – review & editing. **Christoph Jocham:** Writing – review & editing. **Dror Cohen:** Writing – original draft, Writing – review & editing. **Ze’evi Maor:** Writing – review & editing. **Assaf Assis:** Writing – original draft, Writing – review & editing. **Ondrej Panák:** Writing – original draft, Visualisation, Writing – review & editing. **Uroš Novak:** Writing – review & editing. **Sukriti Hans:** Writing – original draft, Writing – review & editing. **Antje Biesemeier:** Writing – original draft, Writing – review & editing. **Pau Camilleri:** Writing – review & editing. **Fruela Pérez Sánchez:** Writing – original draft, Writing – review & editing. **Thomas Arblaster:** Writing – review & editing. **Nils Thonemann:** Writing – review & editing. **Jeroen Guinee:** Writing – review & editing. **Andrea Pipino:** Writing – review & editing. **Onur Çelen:** Writing – review & editing. **Hariprasad Alwe:** Writing – review & editing. **Roland Drexel:** Writing – review & editing. **Roland Welz:** Writing – review & editing. **Florian Meier:** Writing – review & editing. **Indre Piragyte-Langa Oliva:** Writing – review & editing. **Ghada Tagorti:** Writing – review & editing. **Barry Hardy:** Writing – review & editing. **Milica Velimirovic:** Writing – original draft, Writing – review & editing, Conceptualization, Funding acquisition, Supervision.

## Declaration of Competing Interest

The authors declare that they have no known competing financial interests or personal relationships that could have appeared to influence the work reported in this paper.
